# *Dalbergia odorifera* Trans-Nerolidol Protects Against Myocardial Ischemia via Downregulating Cytochrome- and Caspases-Signaling Pathways in Isoproterenol-Induced Rats

**DOI:** 10.3390/ijms26052251

**Published:** 2025-03-03

**Authors:** Canhong Wang, Yulan Wu, Bao Gong, Xiangsheng Zhao, Hui Meng, Junyu Mou, Xiaoling Cheng, Yinfeng Tan, Jianhe Wei

**Affiliations:** 1 Hainan Provincial Key Laboratory of Resources Conservation and Development of Southern Medicine, Hainan Branch of the Institute of Medicinal Plant Development, Chinese Academy of Medical Sciences, Peking Union Medical College, Haikou 570311, China; xinzhuangjianpo@163.com (C.W.); wyl18908698212@163.com (Y.W.); gongbao121@163.com (B.G.); xiangshengzhao@hotmail.com (X.Z.); hmeng@implad.ac.cn (H.M.); mjy5197664@163.com (J.M.); 18162350450@163.com (X.C.); 2 Guangdong Key Laboratory of Green Agricultural Products Processing and Intelligent Equipment, Guangdong University of Petrochemical Technology, Maoming 525099, China; 3 Hainan Provincial Key Laboratory for Research and Development of Tropical Herbs, Hainan Medical University, Haikou 571199, China; 4 Key Laboratory of Bioactive Substances and Resources Utilization of Chinese Herbal Medicine, Institute of Medicinal Plant Development, Chinese Academy of Medical Sciences, Peking Union Medical College, Beijing 100193, China

**Keywords:** *Dalbergia odorifera* trans-nerolol, isoproterenol, myocardial ischemia, cytochrome, anti-apoptosis

## Abstract

*Dalbergia odorifera* is widely used to treat cardiovascular diseases. Our research group found that *Dalbergia odorifera* volatile oil has a good anti-myocardial ischemic effect, and its main pharmacodynamic components are trans-nerolol and its oxides. However, the exact mechanisms underlying this effect have not yet been elucidated. This study aimed to explore the potential myocardial protective effects of trans-nerolol and its underlying molecular mechanisms. Molecular docking was used to predict and visualize the possible mechanism of the anti-apoptotic myocardial protection by trans-nerolol. The myocardial protective effect of trans-nerolol was evaluated by observing pathological injury, myocardial enzyme levels, oxidation, antioxidant levels, and the expression of related proteins. Molecular docking results showed that trans-nerolol binds closely to cytochrome C (Cytc) and apoptosis-related proteins, suggesting that it may play a role in interacting with these target proteins. The results showed that pre-treatment with dose-dependent trans-nerolol significantly mitigated the myocardial histological damage; decreased lactate dehydrogenase (LDH), creatinine kinase (CK), alanine transaminase (ALT), and aspartate transaminase (AST) levels; reduced nitric oxide (NO) production, hydrogen peroxide (H_2_O_2_), and lipid peroxide (LPO); and increased the total antioxidant content (T-AOC), glutathione (GSH), catalase (CAT), and superoxide dismutase (SOD) activities compared with the model group. In addition, dose-dependent trans-nerolol significantly increased the Na^+^-K^+^-ATPase and Ca^2+^-Mg^2+^-ATPase levels. Moreover, trans-nerolol markedly reduced the endogenous and external apoptotic pathways; downregulated the protein expression of Cytc, apoptotic protease activating factor-1 (Apaf1), Fibroblast-associated (Fas), Cysteine-aspartate protease 3 (Caspase3), Cysteine-aspartate protease 8 (Caspase8), and Cysteine-aspartate protease 9 (Caspase9); and upregulated the expression of Heat shock protein 70 (Hsp70) and B-cell lymphoma-2 (Bcl-2). These data indicate that trans-nerolol exerts protective effects against myocardial ischemia (MI), and its mechanism is associated with the suppression of the Cytc- and caspase-signaling pathways. Trans-nerolol has a therapeutic effect on MI, and its mechanism of action is related to its anti-apoptotic effect. These results suggest that *Dalbergia odorifera* has a potential role to be developed as an MI-promoting therapeutic agent.

## 1. Introduction

*Dalbergia odorifera* T. Chen is indigenous to Hainan province [1], and its heartwood is an important traditional Chinese medicine (TCM) named “Jiangxiang” in Chinese, which is used for dissipating blood stasis, regulating the flow of qi, and relieving pain. Moreover, it has been used to treat cardio-cerebrovascular diseases, blood disorders, ischemia, and pain relief in China [2]. Modern pharmacological studies have revealed that *D. odorifera* has various biological effects, such as anti-inflammatory [3,4], antioxidant [5], angiogenic [6], anti-platelet aggregation [7], anti-tumor [8,9] and anti-microbial [7,10] effects. Modern chemical composition studies have shown that volatile oils and flavonoids are the major components of *D. odorifera* [11]. Previous studies by our research group have confirmed that both essential oils and flavonoids of *D. odorifera* have significant anti-myocardial ischemia effects, and that the main pharmacodynamic components of the essential oil were trans-nerol and its oxides, and its mechanism was related to the inhibition of cytochrome C [12,13]. However, whether trans-nerolol has an anti-myocardial ischemic effect, and whether the mechanism of action is related to the down-regulation of the Cytc-signaling pathway, has not been studied. Therefore, we conducted this study to investigate the myocardial protective effect and molecular mechanism of the monomer components of *D. odorifera* to provide reference and guidance for the development and clinical application of *D. odorifera* as a myocardial protective pharmaceutical product.

Studies have confirmed that *D. odorifera* has a good cardiac protection effect and has a good prevention and treatment effect on cardiac diseases, such as myocardial ischemia. As we all know, cardiovascular disease (CVD) is a major health problem. Owing to its incidence, recurrence rate, and mortality, it has attracted increasing attention from medical clinicians and researchers, especially high risk factors such as CHD, hypertension, and stroke [14,15,16]. CHD is the most common cause of myocardial ischemia (MI), and the most fundamental cause of its pathological symptoms is that the blood and oxygen supply from the coronary artery to the myocardium cannot meet the demand for myocardial oxygen [17,18]. The pathogenesis of MI is complex and varied, including free radical accumulation, calcium overload, energy metabolism disorder, inflammatory response, and apoptosis [19,20,21]. Therefore, the treatment of MI remains a vital problem, with no advanced and effective therapeutic regimen as of now [22]. Over the past decades, TCM has shown a potential therapeutic effect on cardiac diseases and shown a good clinical effect, including *D. odorifera* [23,24]. Furthermore, a previous study revealed that essential oils from *D. odorifera* provide significant myocardial protection [12]. Therefore, further experimental studies are required to explore the potential roles and molecular mechanisms of *D. odorifera* components in the treatment of MI.

The present study was designed for the first time to investigate the potential cardioprotective effect of *D. odorifera* trans-nerolol on isoproterenol (ISO)-induced MI in rats and explore its possible mechanisms, including anti-internal and anti-external apoptosis effects. This study provides experimental evidence for further research and clinical treatment of *D. odorifera* against myocardial diseases.

## 2. Results

### 2.1. GC-MS Analysis of Essential Oil Components of D. odorifera

**Figure 1:**
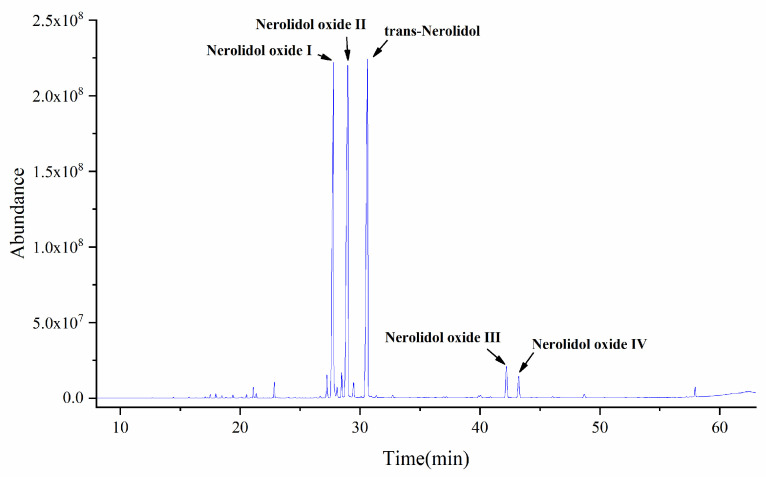
GC-MS total ion flow diagram of *D. odorifera* volatile oil.

**Figure 2:**
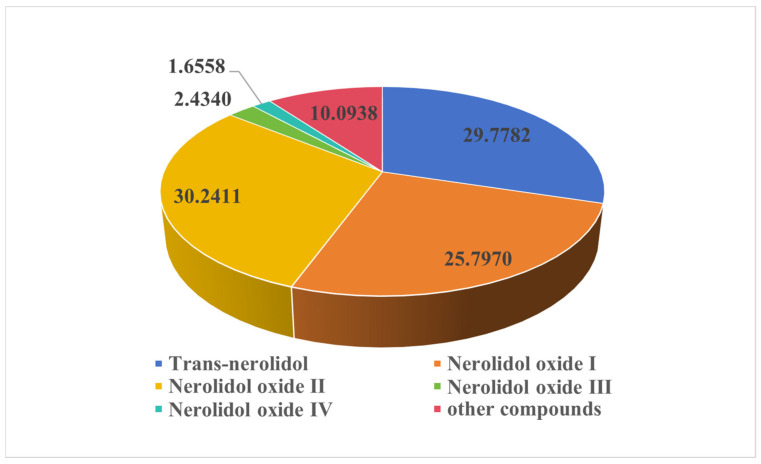
The proportion of components content of *D. odorifera* volatile oil.

A total of 37 compounds were isolated and identified from the essential oil, and the main components were trans-nerol, its oxides, and other components. Among them, the contents of trans-nerol and its oxides were as high as 90%, with trans-nerolidol (29.7782%), nerolidol oxide I (25.7970%), nerolidol oxide II (30.2411%), nerolidol oxide III (2.4340%), nerolidol oxide IV (1.6558%), and other compounds (10.0938%), as detailed in Figure 1 and Figure 2.

### 2.2. Molecular Docking of Anti-Apoptotic Mechanism of Trans-Nerolol

**Figure 3:**
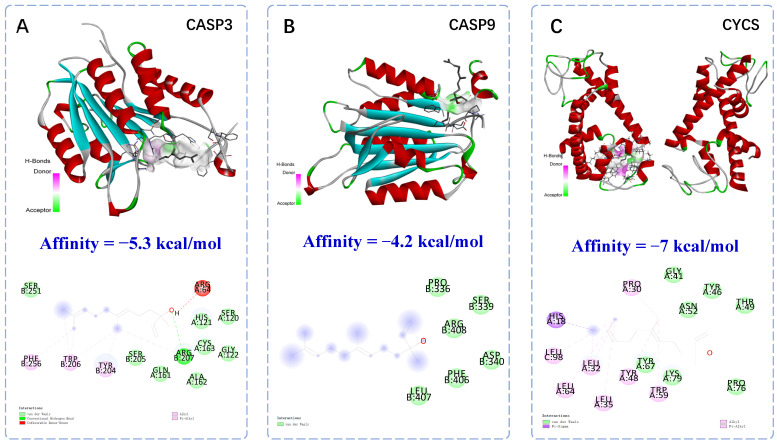

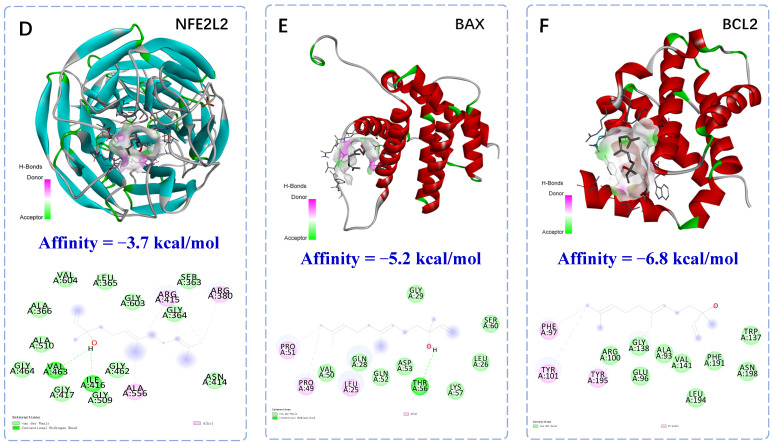
Molecular docking of anti-apoptotic mechanism of trans-nerolol.

Based on molecular docking, trans-nerolol was docked to six proteins. In general, the binding energy is less than −5 kcal/mol, so it is considered that the receptor and ligand could bind well together. Having the lower binding energy, it shows that the receptor and ligand can bind more tightly. The binding site and energy visually showed the stable binding function of the docking model. As shown in Figure 3, the binding energies of trans-nerolol with the five target proteins formed more stable binding conformations with higher values. The core components were closely bound to the target proteins, indicating that these target proteins are key anti-MI targets. Moreover, trans-nerolol was successfully docked to the core objective with specific binding sites, binding distances, and binding atoms, which may involve the formation of hydrogen bonds, van der Waals interactions, and pi-sigma/Alkyl bonds. The structural formula of the compound indicates that trans-nerolol contains hydroxyl, unsaturated double bonds, alkyl substituents, and other free active functional groups, which are easily combined with the dehydration condensation and decarboxylated oxidation of amino acid residues of the compound. The binding between trans-nerolol and its core target is introduced as an example. The complexes of trans-nerolol with CYCS were stabilized by interactions with the amino acid residues HIS-18, LEU-32/35/64/98, TYR-48, and TPR-59 via pi-sigma/alkyl bonds. The complexes of trans-nerolol with CASP3 and BAX were stabilized by interactions with amino acid residues ARG-207 and THR-56 via conventional hydrogen bonds. The complexes of trans-nerolol with CASP9 were stabilized by interactions with the amino acid residue ARG-408 via van der Waals bonds. These molecular-docking results provide a reference for the molecular mechanism of anti-apoptotic action. However, the binding energy of trans-nerolol and NFE2L2 is only −3.7 kcal/mol, and the binding effect is relatively weak. TCMs have multiple components, targets and pathways. In addition, the mechanism of disease occurrence is complex and involves a large number of proteins and only molecular docking with a small number of known proteins. In this study, only the apoptosis and oxidation of individual proteins were interfaced. Therefore, the targeted effects of trans-nerolol and other components of *D. odorifera* should be the focus of future studies.

### 2.3. Effect of Trans-Nerolol on Myocardial Enzymes in Serum

**Figure 4:**
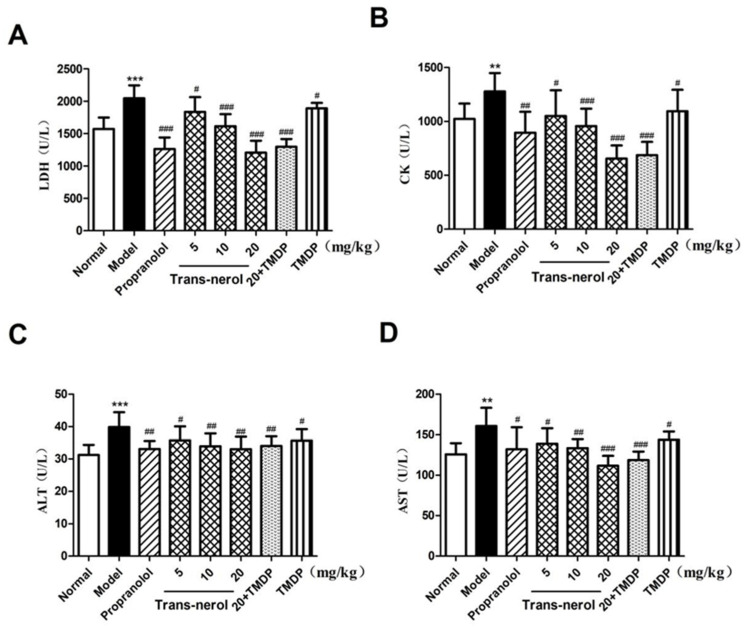
Effects of trans-nerol on myocardial enzymes in homogenate supernatants of heart: (**A**) LDH, (**B**) CK, (**C**) ALT, and (**D**) AST. Each value represents the mean ± SD with n = 6, ** *p* < 0.01, and *** *p* < 0.001 vs. the normal group, # *p* < 0.05, ## *p* < 0.01, and ### *p* < 0.001 vs. the model group.

As shown in Figure 4A–D, LDH, CK, ALT, and AST in the MI group were higher than those in the control group (*p* < 0.01, *p* < 0.001). After treating with trans-nerolol, the concentrations of LDH, CK, ALT, and AST were lower than those in the MI group (*p* < 0.05, *p* < 0.01, *p* < 0.001, respectively). The effects of trans-nerolol were similar to those of propranolol alone; that is, dose-dependent trans-nerolol clearly improved ISO-induced MI. In addition, TMDP decreased the myocardial enzyme levels. Trans-nerolol 20 mg/kg combined with TMDP had a stronger antagonistic effect on myocardial enzymes, suggesting that trans-nerolol acts in cooperation with TMDP, an ISO antagonist.

### 2.4. Effect of Trans-Nerolol on the Lipid Peroxidation in Homogenate Supernatant

**Figure 5:**
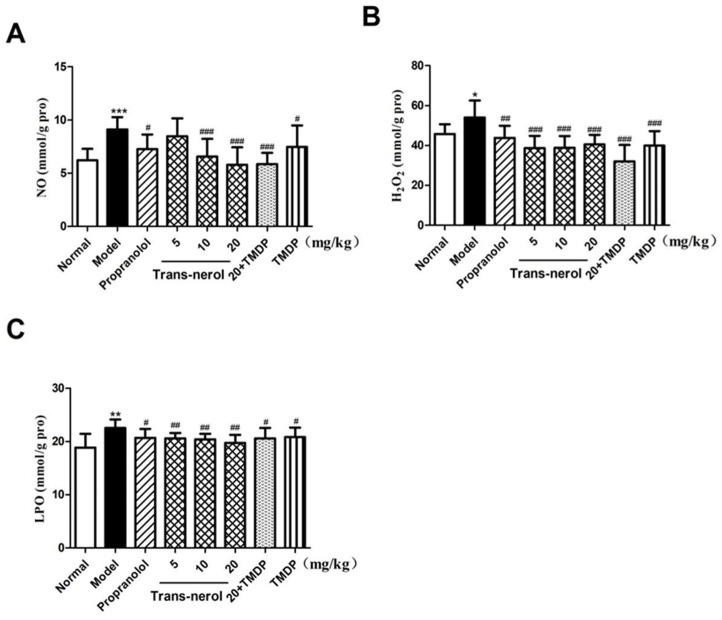
Effects of trans-nerol on the lipid peroxidation in homogenate supernatants of the heart. (**A**) NO, (**B**) H_2_O_2_, and (**C**) LPO. Each value represents the mean ± SD with n = 6, * *p* < 0.05, ** *p* < 0.01, and *** *p* < 0.001 vs. the normal group, # *p* < 0.05, ## *p* < 0.01, and ### *p* < 0.001 vs. the Model group.

As shown in Figure 5A–C, the levels of NO, H_2_O_2_, and LPO were significantly increased (*p* < 0.05, *p* < 0.01, *p* < 0.001, respectively) in rats with MI compared with those in normal rats, confirming that oxidative damage occurred after ISO treatment. However, trans-nerolol markedly decreased the levels of NO, H_2_O_2_, and LPO (*p* < 0.05, *p* < 0.01, *p* < 0.001, respectively) compared to those in the model group. Moreover, TMDP treatment decreased lipid peroxidation. A high dose of trans-nerolol combined with TMDP inhibited lipid peroxidation to a greater extent.

### 2.5. Effect of Trans-Nerolol on the Anti-Oxidation in Homogenate Supernatant

**Figure 6:**
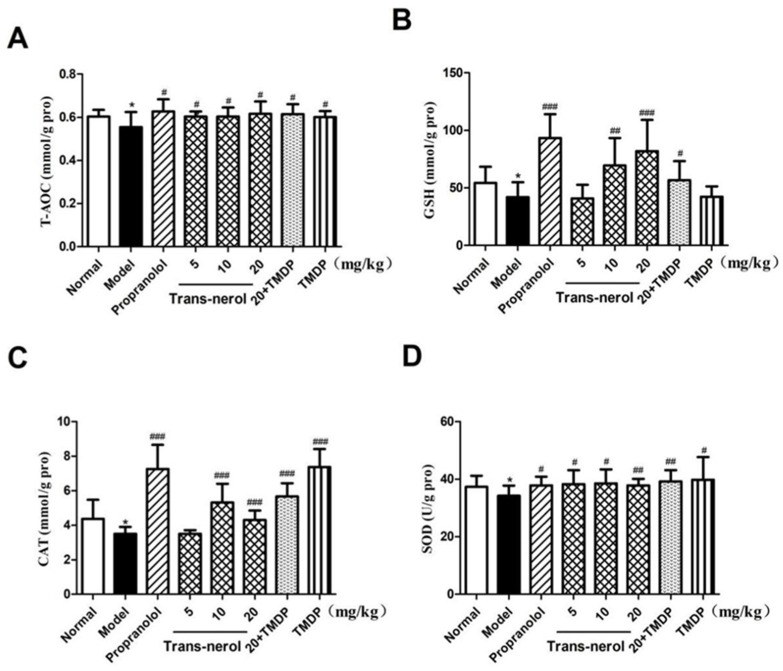
Effects of trans-nerol on the lipid peroxidation in homogenate supernatant of heart: (**A**) T-AOC, (**B**) GSH, (**C**) CAT, and (**D**) SOD. Each value represents the mean ± SD with n = 6, * *p* < 0.05 vs. the normal group, # *p* < 0.05, ## *p* < 0.01, and ### *p* < 0.001 vs. the model group.

As shown in Figure 6A–D, the levels of T-AOC, GSH, CAT, and SOD were significantly decreased (*p* < 0.05) in rats with MI compared to those in normal rats. However, the levels of T-AOC, GSH, CAT, and SOD were significantly increased in trans-nerolol and positive groups (*p* < 0.05, *p* < 0.01, *p* < 0.001) compared to the model group, showing the potential anti-oxidative effect of trans-nerolol. Moreover, TMDP also increased the levels of the anti-oxidation. Trans-nerolol 20 mg/kg combined with TMDP had a greater effect on the anti-oxidation than the TMDP alone.

### 2.6. Effect of Trans-Nerolol on the ATPase in Homogenate Supernatant

**Figure 7:**
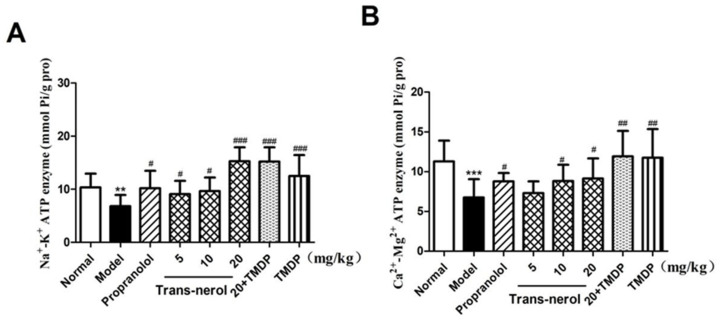
Effects of trans-nerol on the ATPase in homogenate supernatant of heart. (**A**) Na^+^-K^+^-ATPase, (**B**) Ca^2+^-Mg^2+^-ATPase. Each value represents the mean ± SD with n = 6, ** *p* < 0.01 and *** *p* < 0.001 vs. the normal group, # *p* < 0.05, ## *p* < 0.01 and ### *p* < 0.001 vs. the model group.

As shown in Figure 7A,B, the levels of Na^+^-K^+^-ATPase and Ca^2+^-Mg^2+^-ATPase were significantly decreased (*p* < 0.01, *p* < 0.001) in rats with MI, compared with those in normal rats, confirming that the myocardial metabolic function decreased significantly after ISO treatment. However, trans-nerolol markedly increased the levels of Na^+^-K^+^-ATPase and Ca^2+^-Mg^2+^-ATPase (*p* < 0.05, *p* < 0.01, *p* < 0.001) compared to those in the model group, showing that trans-nerolol improved the myocardial function. In addition, TMDP increased the ATPase. A high dose of trans-nerolol combined with TMDP increased the ATPase activity to the greatest extent.

### 2.7. Myocardium Histopathological Examination

**Figure 8:**
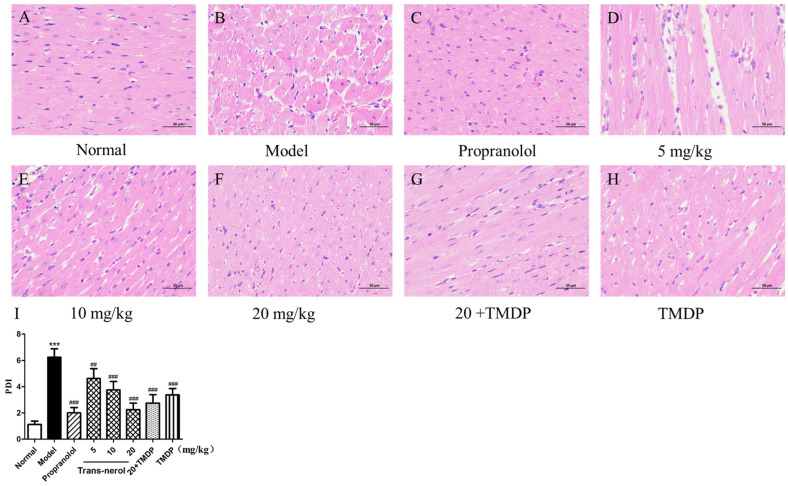
Effects of trans-nerol on the myocardium histopathological examination of the heart: (**A**) normal group, (**B**) model group, (**C**) propranolol group, (**D**) 5 mg/kg group, (**E**) 10 mg/kg group, (**F**) 20 mg/kg group, (**G**) 20+TMDP group, (**H**) TMDP group and (**I**) Pathological damage index(PDI). Each value represents the mean ± SD with n = 3, *** *p* < 0.001 vs. the normal group, ## *p* < 0.01, ### *p* < 0.001 vs. the model group.

To observe the degree of damage to the cardiac tissues, macroscopic myocardial biopsies were performed as shown in Figure 8. In the normal group, a thin and compact arrangement of striated muscle tissue was observed in the heart, as determined by HE staining (Figure 8A). However, in the cardiac tissues of the MI group, most muscle fibers in the striated muscle tissue showed dissolution, muscle fiber disorder, edema, degeneration, necrosis, inflammatory cell infiltration, numerous foam cells, hyperemia, and bleeding (Figure 8B). The above damage in the MI rats treated with trans-nerolol improved compared to that in the MI group (Figure 8C–H). The histopathological injury index histogram displays the results more intuitively (Figure 8I). The pathological damage index of the model group was more than three times that of those in the normal group, and the pathological damage index of the high-dose treatment group was less than half that of the model group. The effect of a high dose of trans-nerolol was similar to that of the positive drugs. The combination of high levels of trans-nerolol and TMDP enhanced the protective effects of TMDP against myocardial pathological injury.

### 2.8. Effect of Trans-Nerolol on the Protein Expressions of Endogenous Apoptotic Pathway by IHC

**Figure 9:**
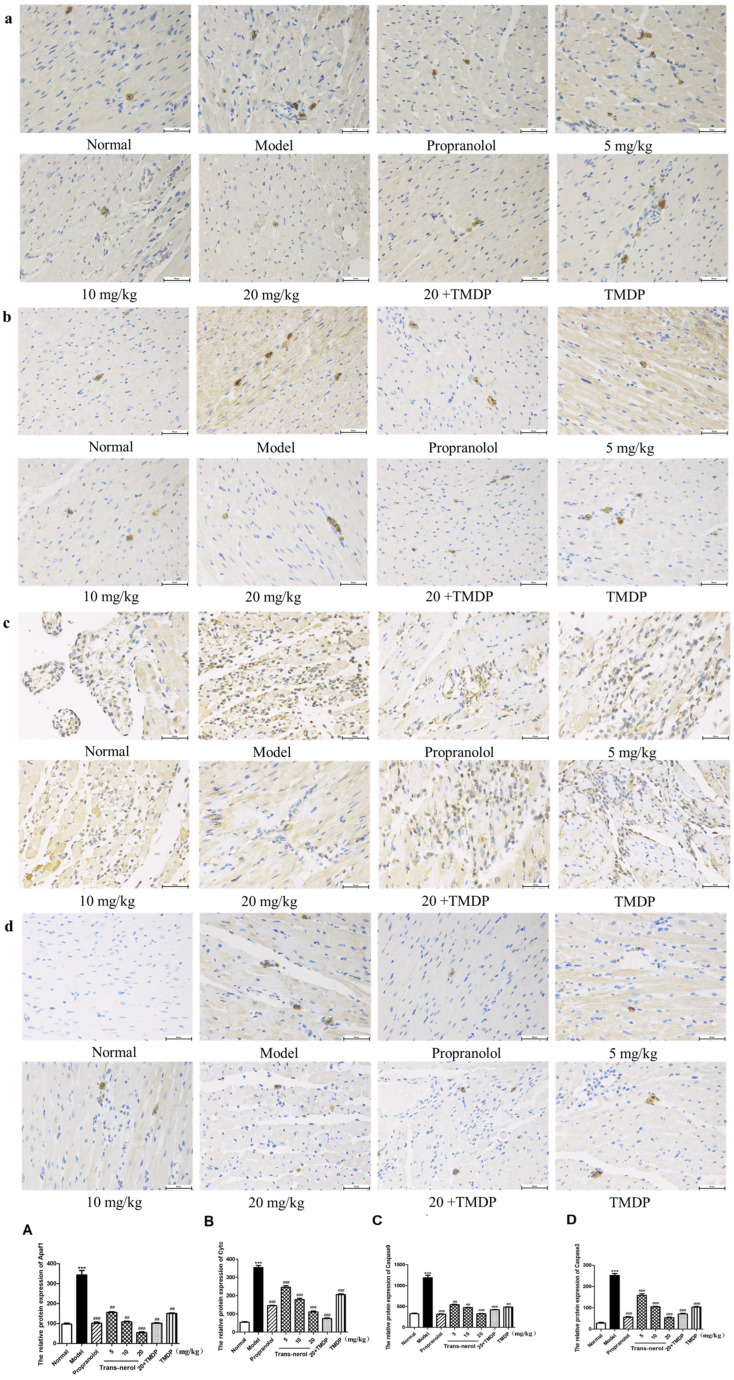
Effects of trans-nerol on the protein expressions of the endogenous apoptotic pathway in the heart tissue: (**a**) Apaf1, (**b**) Cytc, (**c**) Caspase9, and (**d**) Caspase3. (**A**–**D**) Scale bar = 50 μm. Relative protein expression. Each value represents the mean ± SD with n = 3, *** *p* < 0.001 vs. the normal group, ## *p* < 0.01, ### *p* < 0.001 vs. the model group.

To explore the protective mechanisms mediated by trans-nerolol anti-apoptosis in MI induced by ISO, we further detected the protein expression of the endogenous apoptotic Cytc pathway in the heart tissue by IHC. As shown in Figure 9a–d, treatment with trans-nerolol significantly decreased the protein expression levels of Apaf1, Cytc, Caspase9, and Caspase3 in the heart tissue of ISO-induced MI rats. As shown in Figure 9A–D, the expression of apoptosis-related proteins in the model group was more than four times that in the normal group. The expression of apoptosis-related proteins in the model group was reduced dose-dependently by trans-nerolol; especially, the expression of a high-dose-treatment group was less than one-third of that in the model group, suggesting that trans-nerolol has a significant anti-apoptosis damage effect by down-regulating and inhibiting the Cytc pathway.

### 2.9. Effect of Trans-Nerolol on the Protein Expressions of Exogenous Apoptosis Pathway by IHC

**Figure 10:**
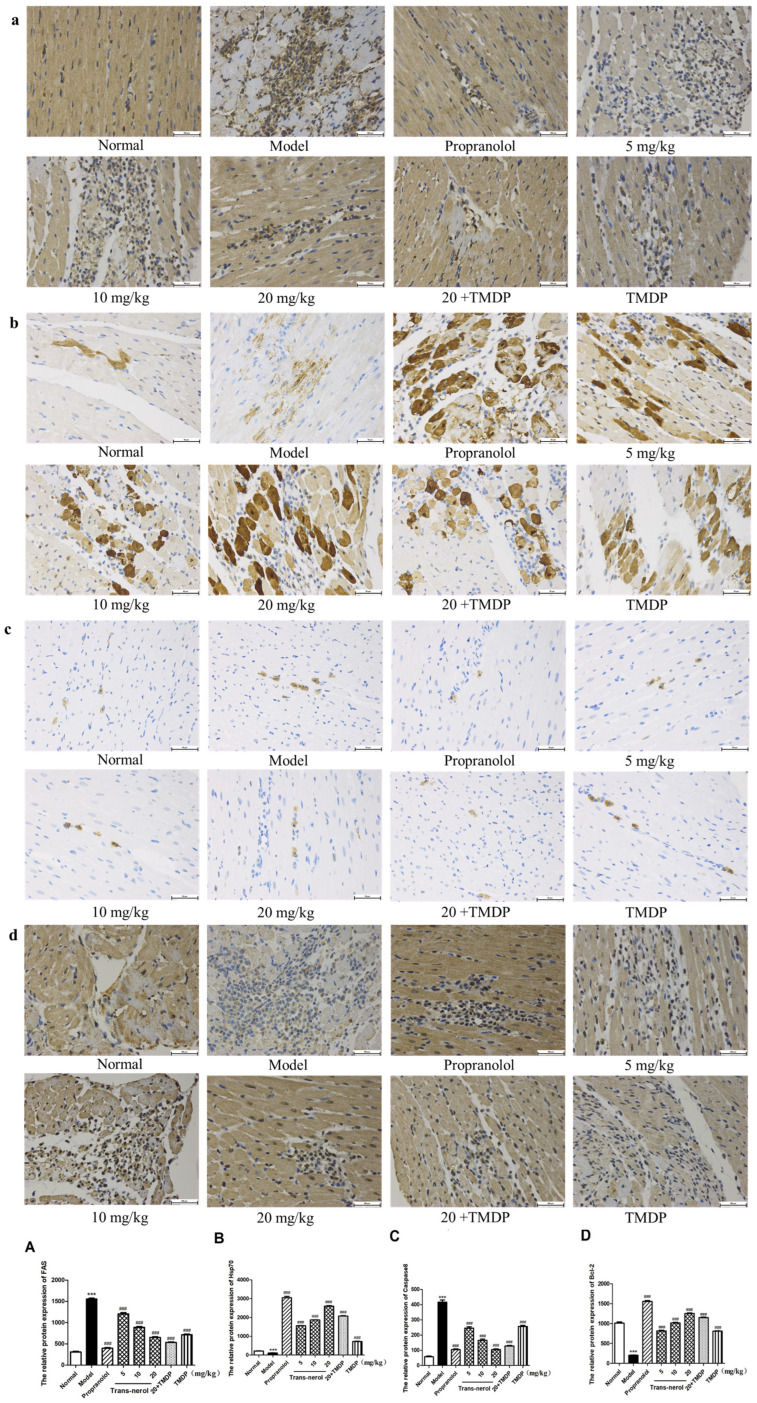
Effects of trans-nerol on the protein expressions of exogenous apoptosis pathway in the heart tissue: (**a**) Fas, (**b**) Hsp70, (**c**) Caspase8, and (**d**) Bcl-2. (**A**–**D**) Scale bar = 50 μm. Relative protein expression. Each value represents the mean ± SD with n = 3, *** *p* < 0.001 vs. the normal group, ### *p* < 0.001 vs. the model group.

Next, to explore the protective mechanisms mediated by trans-nerolol against exogenous apoptosis in MI induced by ISO, we also detected the protein expression of the caspase pathway by IHC in heart tissue. As shown in Figure 10a–d, it is particularly gratifying that trans-nerolol markedly decreased the protein expression levels of Fas and Caspase8 and increased Hsp70 and Bcl-2 in the heart tissue. As shown in Figure 10A,C, the expression of apoptosis-related proteins Fas and Caspase8 in the model group was more than four times that in the normal group. The expression of apoptosis-related proteins in the model group was reduced dose-dependently by trans-nerolol; especially, the expression of a high-dose-treatment group was less than one-two of that in the model group. As shown in Figure 10B,D, compared with the normal group, the expressions of anti-apoptosis-related proteins Hsp70 and Bcl-2 in the model group were significantly reduced, less than half of those in the normal group. However, trans-nerol increased their expression in a dose-dependent manner, especially a high-dose group by a factor of ten and five, respectively, showing a significant anti-apoptotic effect by regulating the caspase pathway.

### 2.10. Effect of Trans-Nerolol on the Protein Expressions of Apoptosis Pathways by WB

**Figure 11:**
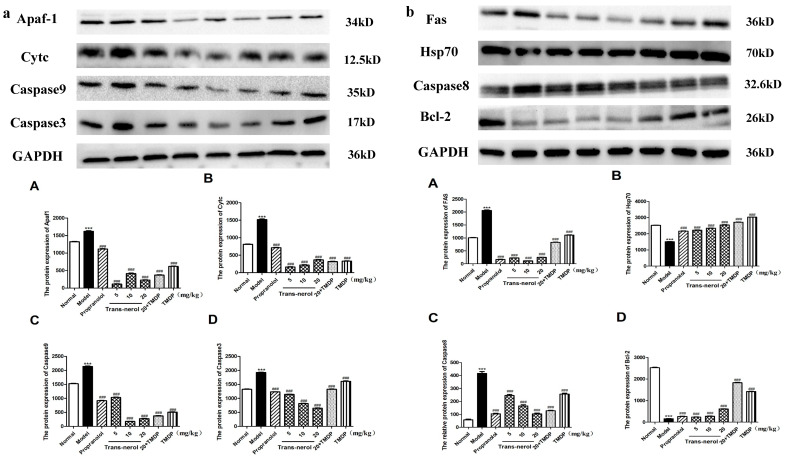
Effects of trans-nerol on the protein expressions of apoptosis pathways in the heart tissue: (**a**) Apaf1, Cytc, Caspase9 and Caspase3. (**b**) Fas, Hsp70, Caspase8, and Bcl-2. (**A**–**D**) Relative protein expression of Figure a and b, respectively. Each value represents the mean ± SD with n = 3, *** *p* < 0.001 vs. the normal group, ### *p* < 0.001 vs. the model group.

Additionally, we detected the protein expressions of endogenous and exogenous apoptotic pathways in MI rats by WB. As shown in Figure 11a,b, trans-nerolol markedly reduced the protein expression levels of Apaf1, Cytc, Caspase3, Caspase9, Fas, and Caspase8; compared to the model group, the reduction is at least three times and up to ten times. On the contrary, trans-nerolol significantly increased the expressions of the Hsp70 and Bcl-2, showing a significant anti-apoptotic effect by down-regulating the Cytc and caspase pathways.

## 3. Discussion

**Figure 12:**
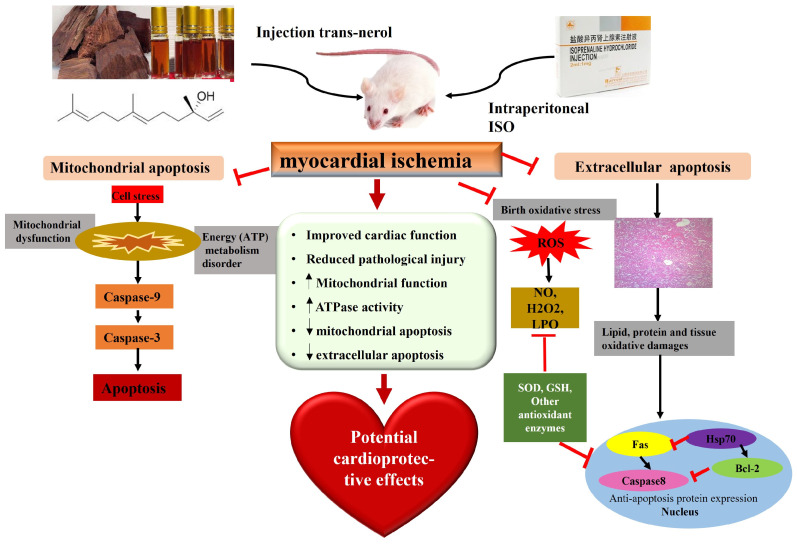
The mechanism of action of trans-nerolol against myocardial ischemia. Black arrows indicate activation, red arrows indicate inhibition.

*D. odorifera*, a precious and aromatic southern medicine in China, has been used for centuries to treat several diseases, especially heart disease [25,26]. A literature search revealed that *D. odorifera* has been used in more than 100 TCMs, such as Qi Shen Yi Qi pills, Guanxin Danshen Pills, Xiang Dan injection, and Tongxinluo capsules [27,28,29], but no single drug for *D. odorifera* has been developed to date. Studies have shown that a compound preparation containing the volatile oil of *D. odorifera* has good therapeutic effects on CHD [23,25,26,27,28]. Few studies have revealed that volatile oils are the main components of *D. odorifera* [30]. Our study showed that the volatile oil of *D. odorifera* has a significant therapeutic effect on MI. However, there have been no studies on the anti-myocardial ischemic effects of *D. odorifera* trans-nerolidol. This study aimed to evaluate the pharmacological effects and mechanisms of the trans-nerolol of *D. odorifera* against MI. In the present study, we examined ISO-induced MI in rats and found that trans-nerolidol protected against MI, markedly inhibited oxidative stress, and exerted anti-apoptotic effects, alleviating ISO-induced MI injury. This mechanism may be associated with the regulation of endogenous and exogenous apoptotic pathways, as shown in Figure 12.

The injury characteristics of MI include changes in the myocardial tissue cell structure, myocardial remodeling, and systolic and diastolic dysfunction. Abnormal leakage of serum myocardial enzymes is an important marker of myocardial cell membrane injury and increased levels of LDH, CK, ALT, and AST often used in the diagnosis and monitoring of MI [31]. Pathological myocardial injury and myocardial enzyme levels were significantly elevated in the model group. Trans-nerolol reduced the degree of pathological injury caused by MI and decreased myocardial enzyme levels, suggesting that trans-nerolol has an obvious myocardial protective effect.

Antagonizing oxidative stress is an important mechanism in myocardial ischemia [32]. During myocardial ischemic injury, a large number of free oxygen radicals are produced, such as NO, H_2_O_2_, LPO, etc. [33]. However, oxygen free radicals are eliminated by antioxidant systems, such as T-AOC, GSH, CAT, and SOD [34]. Our results showed that trans-nerolol enhanced the levels of GSH, CAT, and SOD and decreased the contents of NO, LPO, and H_2_O_2_. Trans-nerolol had a significant effect on free radical scavenging and anti-oxidative damage, indicating a trans-nerolol-mediated antioxidant effect.

ATPases play an important role in cardiomyocyte apoptosis and myocardial function metabolism during myocardial ischemic injury. The reduction in Na^+^/K^+^-ATPase activity in the cardiac tissue induces myocyte death and cardiac dysfunction, leading to the development of myocardial dilation and myocardial injury in animal models [35,36]. A decrease in Ca^2+^-Mg^2+^-ATPase levels is closely associated with the occurrence and development of myocardial apoptosis. Moreover, calcium homeostasis plays an important role in maintaining mitochondrial and endoplasmic reticulum functions. MI injury is accompanied by intracellular Ca^2+^ overload, which activates the endogenous apoptotic Cytc signaling pathway and contributes to myocardial apoptosis and death [37,38]. Our study suggests that trans-nerolol significantly increases the levels of ATPases, contributing to the anti-apoptotic effect of MI by controlling the ATPase decrease and restoring cardiac function.

Several studies have shown that endogenous and exogenous apoptosis plays an important role in the occurrence and development of myocardial ischemia, especially in the caspase family and Cytc signaling pathway [39,40,41,42]. Previous studies have also confirmed that the essential oil can play an anti-myocardial ischemia role by down-regulating the expression of caspase-family-related proteins and Cytc by our research group [13,14]. Trans-nerolol is the main pharmacodynamic component of essential oils, and we speculate that it also delivers myocardial protection through an anti-apoptotic mechanism. The molecular-docking results also showed that trans-nerolol could bind closely to endogenous- and exogenous-apoptosis-related proteins. To further determine its anti-apoptotic mechanism, this study showed that trans-nerolol significantly reduced the expression of apoptosis-related proteins, contributing to the anti-apoptotic effect of MI by inhibiting apoptosis in heart tissues.

This study confirmed that ISO induced myocardial ischemia in rats, resulting in insufficient blood supply, ischemic damage, and abnormal increase in myocardial enzymes. A stress reaction occurs, and excessive release of oxidative free radicals further aggravates the damage of the myocardium when the heart is damaged. The heart muscle is damaged, resulting in an abnormal decrease in ATPase activity, and the heart cannot function properly. This further aggravates the damage of the heart, appeared pathological damage, apoptosis, and necrosis. The occurrence of cardiac apoptosis is regulated by apoptosis-related signaling pathways, the occurrence of apoptosis activates the caspase family and Cytc pathway, induced the overexpression of apoptosis-related proteins, and the physiological response of cardiac apoptosis occurs. Trans-nerolol antagonized ISO-induced myocardial enzyme, peroxidation index, pathological injury, and apoptosis-related protein expression in a dose-dependent manner and significantly reduced ISO-induced cardiac ischemic injury. These results are consistent with previous research [12,13,14,23].

## 4. Materials and Methods

### 4.1. Materials

#### 4.1.1. Reagents

ISO was obtained from Harvest Pharmaceutical Co., Ltd. (Shanghai, China). Myocardial enzymes lactate dehydrogenase (LDH), creatinine kinase (CK), alanine transaminase (ALT), and aspartate transaminase (AST) were purchased from Shanghai Coaibo Biotechnology Co., Ltd. (Shanghai, China). All biochemical indicator kits, hydrogen peroxide (H_2_O_2_), nitric oxide (NO), lipid peroxide (LPO), total antioxidants contents (T-AOC), glutathione (GSH), catalase (CAT), superoxide dismutase (SOD), sodium–potassium ATPase (Na^+^-K^+^-ATPase), and calcium–magnesium ATPase (Ca^2+^-Mg^2+^-ATPase) were derived from Jian Cheng Biotech Co. (Nanjing, China). The Cytc and caspase pathways were purchased from Abcam (Cambridge, UK). Anhydrous alcohol and other chemicals were of analytical grade.

#### 4.1.2. Drugs

**Figure 13:**
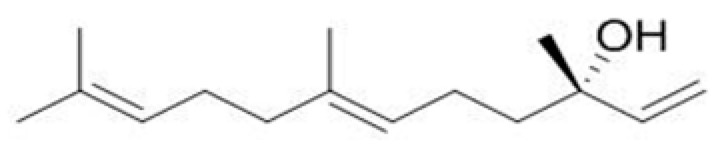
Structural formula of trans-nerol.

The trans-nerolol (Purity greater than 95%) was bought from Shanghai Yuanye Biotechnology Co., Ltd. (Shanghai, China) (Figure 13). Nerolidol (molecular formula: C15H26O, molecular weight: 222.37) is a naturally occurring sesquiterpene found in essential oils of many plants and flowers types. The positive control drug, propranolol, was purchased from Shanghai Aladdin Biochemical Technology Co., Ltd. (Shanghai, China). 2-chloro-4,4,5, 5-tetramethyl-1,3, 2-dioxyphosphoheterocyclopentane (TMDP) was purchased from Shanghai Maclin Biochemical Technology Co., Ltd. (Shanghai, China). All these drugs were stored in the refrigerator (4 °C).

### 4.2. Methods

#### 4.2.1. Detection and Analysis of Essential Oil by GC-MS

##### GC-MS Analysis Condition

Agilent HP INNOWax Capillary column (30 m × 0.25 mm, 0.25 μm); temperature program: starting temperature 100 °C, maintain 2 min, 3 °C·min^−1^ temperature rise to 160 °C, 1 °C·min^−1^Heat up to 190 °C at 5 °C·min^−1^ Heating up to 220 °C, At 10 °C·min^−1^. Temperature rise to 250 °C; Inlet temperature 230 °C; Shunt ratio 10:1; Sample size 1 μL; The carrier gas is helium; The flow rate is 1.0 mL·min^−1^. The temperature of EI ion source is 230 °C. Transmission line temperature 250 °C; Ionization voltage 70 eV; Quality scanning range *m*/*z* 50–600, solvent delay 8 min.

##### Preparation and Composition Detection of Volatile Oil

The volatile oil was extracted and dehydrated with an appropriate amount of anhydrous sodium sulfate. In total, 10 μL of the volatile oil was dissolved in 1 mL in the ethyl acetate, over a 0.22 μm filter membrane, and set aside. The volatile components of the volatile oil were analyzed via GC-MS, and the total ion flow diagram (Figure 1) was obtained. The detected components were qualitatively determined by NIST database matching, some compounds were compared, according to standard substances, and references were made to relevant studies. Peak area normalization method was used to calculate the relative content (%) of each compound, as shown in Table S1.

#### 4.2.2. Component–Target Molecular Docking

Molecular docking was used to identify the binding affinity between trans-nerol and target proteins (Table 1), which provided a reference for predicting the pharmacodynamic mechanism. AutoDock Vina 1.1.2 is an open-source program for molecular docking and virtual screening, which provides a high average accuracy value of binding-mode predictions for docking experiments. The three-dimensional structures of the target proteins were downloaded from the Protein Data Bank (PDB) database. The ligands and receptors for the molecular-docking analysis were prepared using AutoDock Vina 1.1.2. Water molecules were deleted from each structure, nonpolar hydrogen atoms were added, and the Gasteiger charge was calculated and saved in the PDBQT format. The lower the Vina score, the higher is the affinity between the components and proteins. Usually, the lowest affinity is regarded as the best docking, and the visual interaction mode is shown by PLIP.

#### 4.2.3. Animal Experiments

Forty-eight rats were randomly divided into normal, ISO, propranolol (20 mg/kg), trans-nerolol low dose (5 mg/kg), trans-nerolol medium dose (10 mg/kg), trans-nerolol high dose (20 mg/kg), trans-nerolol high (20 mg/kg) +TMDP (5 mg/kg), and TMDP (5 mg/kg) groups (dosage selection basis: according to the pre-test and reference to the use of other natural compound monomers). Rats in the normal and ISO groups were orally administered distilled water (20 mL/kg, 1 time/day). Rats in the positive and trans-nerolol treatment groups were intraperitoneally injected (10 mL/kg, 1 time/day) for seven consecutive days. Rats were subcutaneously injected with ISO (2 mg/kg and 1 mg/kg), except for the normal group, on two consecutive days to establish the MI model [24]. Animal experiments were performed at the Hainan Pharmaceutical Research Institute Co., Ltd. (Haikou, China), approval: 2023HL013.

#### 4.2.4. Serum and Heart Tissue Homogenate Supernatant Preparation

Rats were anesthetized with pentobarbital sodium (concentration: 10 mg/mL, volume: 0.3 mL/100 g, dose: 30 mg/kg). The animals weighed approximately 250 g at the time of euthanasia. Blood samples (5 mL) were collected from the abdominal aorta. The serum was prepared by centrifugation and stored at 4 °C. Euthanasia was inducted by cervical dislocation, and death was verified by the absence of breathing.

The heart tissue was removed, weighed, and saline was added at a ratio of 1:9 (M:V). The mixture was thoroughly homogenized and centrifuged at 4 °C, 3000 rpm and 15 min, and the homogenate supernatant was stored at −80 °C.

#### 4.2.5. Levels of Myocardial Enzymes in Homogenate Supernatant of Heart Tissue

Myocardial cellular damage was evaluated by measuring the myocardial enzymes in the homogenate supernatant of the heart tissue. The levels of LDH, CK, ALT, and AST were measured according to the manufacturer’s instructions.

#### 4.2.6. Determination of Oxidation Index Level in Homogenate Supernatant of Heart Tissue

The heart tissue was clipped and weighed, and normal saline was added at a ratio of 1:9 (M:V) and centrifuged at 3000 rpm at 4 °C for 15 min. The homogenate supernatant was collected and stored at −80 °C. LPO, NO, H_2_O_2_, T-AOC, GSH, CAT, and SOD were detected using special kits, following the manufacturer’s instructions.

#### 4.2.7. Determination of ATP Level in Homogenate Supernatant of Heart Tissue

Blood was collected and centrifuged at 3000 rpm at 4 °C for 15 min. The homogenate supernatant of heart tissue was collected and stored at −80 °C. Na^+^/K^+^-ATPase and Ca^2+^/Mg^2+^-ATPase were detected using special kits, following the manufacturer’s instructions.

#### 4.2.8. Histopathological Examination of Myocardium

Hearts were removed, fixed in 4% paraformaldehyde, dehydrated, and paraffin-embedded. Next, the samples were sectioned at 5 µm (Leica DMR, Wetzlar, Germany). For histological analysis, sections were stained with hematoxylin and eosin (HE) using standard techniques and examined by light microscopy (Nikon, Tokyo, Japan) at 200× magnification. Histological damage was calculated using the following scoring system (LDI score): muscle fibers in the striated muscle tissue showing dissolution (score: 0–3), degeneration (score: 0–4), necrosis (score: 0–4), and inflammatory cell infiltration (score: 0–3). The levels of proteins involved in antioxidation and apoptosis were analyzed via immunohistochemistry (IHC).

#### 4.2.9. Analyzing the Levels of Proteins Involved in Internal and External Apoptosis by IHC

The protein levels of the anti-oxidation and apoptotic protein levels were determined by IHC. Heart tissues were fixed in formalin solution, dehydrated with gradually increasing concentrations of ethanol, embedded in paraffin, and sectioned. The 5 μm sections were treated with a buffered blocking solution (3% bovine serum albumin in phosphate-buffered saline (PBS) for 15 min. The sections were co-incubated with a primary antibody against Cytc, Apaf1, Fas, Caspase3, Caspase8, Caspase9, Hsp70, and Bcl-2 at a dilution of 1:50 in PBS (*v*/*v*) at 4 °C overnight, followed by washing with PBS, and co-incubation with a secondary antibody at a dilution of 1:500 in PBS (*v*/*v*) at room temperature for 1 h. Thereafter, sections were washed with Tris-HCl (0.05 M, pH 7.66) and co-incubated with a 3,3′-diaminobenzidine solution in darkness at room temperature for 10 min. The sections were washed with Tris-HCl, stained with hematoxylin according to standard protocols, and observed under a light microscope. Image-ProPlus software (version 6.0, Media Cybernetics, Rockville, MD, USA) was used to analyze protein expression.

#### 4.2.10. Analysis the Levels of Proteins Involved in Internal and External Apoptosis by WB

The heart tissues were homogenized in a standard RIPA buffer supplemented with a cocktail of protease and phosphatase inhibitors. The homogenate was then centrifuged at 15,000× *g* at 4 °C for 10 min. The protein concentration was determined using a BCA Protein Assay Kit. According to the molecular weight of the target protein, the appropriate concentration of separation gel was selected, the sample amount was brought to 20 μL and electrophoresed, and then the proteins were transferred to a PVDF membrane and blocked with 5% skim milk powder for 1 h. The primary antibodies used were as follows: Apaf1, Cytc, Caspase3, Caspase9, Fas, Caspase8, Hsp70, and Bcl-2 (1:500); they were added, respectively. GAPDH (1:1000) was added. The membranes were incubated overnight at 4 °C, and the HRP-coupled secondary antibody (1:2000) was incubated with the membrane at room temperature for 2 h. ECL chemiluminescence was performed with a gel imager. Gel-Pro Analyzer 7.0 was used for grayscale scanning and quantitative analysis, and intuitive histograms were produced.

#### 4.2.11. Statistical Analysis

Data were expressed as means ± SEM for eight animals in each group and statistically evaluated using the SPSS17 software (Chicago, IL, USA). Differences between groups were analyzed using the one-way analysis of variance (ANOVA) and Tukey’s post hoc test (TTEST). *p* values of less than 0.05 were considered to indicate statistical significance.

## 5. Conclusions

In this study, our results confirmed the myocardial protection of trans-nerolol in MI, with an effect similar to that of the positive control drug propranolol. From these results, we concluded that the potential mechanisms are related to the antagonism of exogenous apoptosis and mitochondrial apoptosis. The pathogenesis of myocardial ischemia is complex and diverse, and other mechanisms underlying myocardial protective effects require further investigation. At present, most drugs containing *D. odorifera* are compound preparations for the treatment of heart disease, and monomer drugs have not been reported or used. The monomeric compound, trans-nerolol, has a good myocardial protective effect, providing a reference for the development of new monomeric drugs. Our study suggests that *D. odorifera* could be a potential protective drug for the prevention and treatment of cardiac diseases.

## Figures and Tables

**Table 1:** The gene information of the target proteins.

No	Name	Gene Name	Gene Symbol
1	Nrf2	*NFE2L2*	Nfe2l2 nuclear factor, erythroid derived 2, like 2
2	Cytochrome C	*Cycs*	cytochrome c, somatic
3	Caspase9	*Casp9*	caspase 9
4	Caspase3	*Casp3*	caspase 3
5	Bax	*BAX*	BCL2 associated X, apoptosis regulator
6	Bcl-2	*Bcl2*	B cell leukemia/lymphoma 2

## Data Availability

The data supporting the findings of this study are available from the first or the corresponding author upon reasonable request.

## Supplementary Materials

The supporting information can be downloaded at https://www.mdpi.com/article/10.3390/ijms26052251/s1.

## Abbreviations

LDH: decreased lactate dehydrogenase; CK: creatinine kinase; ALT: alanine transaminase; AST: aspartate transaminase; NO: nitric oxide; H_2_O_2_: hydrogen peroxide; LPO: lipid peroxide; T-AOC: total antioxidants contents; GSH: glutathione; CAT: catalase; SOD: superoxide dismutase; Cytc: Cytochrome; TCM: traditional Chinese medicine; CHD: coronary heart disease; MI: myocardial ischemia; ISO: Isoproterenol; Na^+^-K^+^-ATPase: sodium–potassium ATPase; Ca^2+^-Mg^2+^-ATPase: calcium–magnesium ATPase; PDB: Protein Data Bank; HE: hematoxylin–eosin; IHC: immunohistochemistry; TTEST: Tukey’s Post Hoc test.
